# Assessment of the Availability and Accuracy of Dosing Devices Packaged with Oral Liquid Medications in the Ho Municipality of Ghana

**DOI:** 10.1155/2022/9223858

**Published:** 2022-10-20

**Authors:** Yussif Saaka, David M. Nyamadi, Hilda Amekyeh, Adelaide Mensah

**Affiliations:** Department of Pharmaceutics, School of Pharmacy, University of Health and Allied Sciences, PMB 31, Ho, Ghana

## Abstract

**Introduction:**

Administering the right dose of medications is essential in avoiding potentially life-threatening adverse drug reactions. Industry guidelines for manufacturers of oral, over-the-counter, and liquid medications recommend including dose-delivery devices with packaging to limit dosing inaccuracy. This study describes the prevalence and accuracy of dosing devices packaged with oral liquid medications in the Ho municipality of Ghana.

**Methods:**

Dosing device accuracy was determined after deviation of the measured volume from the expected volume was evaluated using the United States Pharmacopoeia criteria.

**Results:**

A total of 78.6% of the oral liquid medications were packaged with a dosing device. The most common dosing devices were cups (83.6%), followed by spoons (14.3%), droppers (1.4%), and syringes (0.7%). The volumes measured with cups (5.14 ± 0.52 mL, *p* = 0.006) and spoons (5.3 ± 0.67 mL, *p* < 0.001) were significantly different from the desired 5 mL volume; this was dissimilar to the volume measured using syringes (5.01 ± 0.02 mL, *p* > 0.999). Further, the measured volumes for 38.6% and 72.2% of the cups and spoons, respectively, deviated by more than 15% of 5 mL.

**Conclusion:**

Dosing cups and spoons are associated with significant inaccuracy. Yet, manufacturers continually favour them over syringes in packaging for oral liquid medications. This is unacceptable and of considerable concern due to the risk of variations in therapeutic outcomes. Therefore, strict regulatory directives on the inclusion of accurate dosing devices in the packaging of oral liquid medicines are needed to reduce the possibility of medication errors.

## 1. Introduction

Overdose of liquid medications has been identified as having severe and potentially fatal consequences, especially in paediatric patients. Liquid medication overdose in paediatric patients results in over 70,000 visits to the emergency room yearly in the United States of America (USA) [1]. Studies demonstrate that the root cause of this high incidence is user errors in dose measurement and dosing device errors [2, 3]. In the USA alone, one child receives a wrong medication dose in a household every 8 minutes [4, 5]^.^ In an attempt to determine the availability and accuracy of dosing devices, Bayor et al. [6] found that dosing devices were not packaged with 46% and 97% of orthodox and herbal oral liquid medicines, respectively, on the Ghanaian market. A similar trend was found in the research by Johnson and Meyers [7] where it was revealed that only 12.8% of 382 liquid prescription medications were packaged with a dosing device. Consequently, caregivers are compelled to use devices such as household spoons to deliver required medication doses. Household spoons have, however, been shown to be inaccurate; their use as measures has been referred to as anachronistic and should no longer be recommended [6, 8, 9].

In response to this, regulatory agencies have developed guidelines recommending the inclusion of measuring devices in over-the-counter (OTC) liquid medications, particularly those intended for use in children [10–13]. A study assessing compliance of OTC liquid products with these guidelines showed a glaring need for improvements in dosing devices for these products, with 98.6% of products having a discrepancy between the dose on the label and the measuring device in the package [10]. There are reports of adherence to the recommendations [14]; however, dosing device availability remains woefully inadequate as it is still not a standard practice for dosing tools to be provided in the clinical or community pharmacy setting [7, 15]. Further, the provision of dosing devices for prescription medications is inadequate compared to OTC medications. Therefore, in recent times, the National Council for Prescription Drug Programs published a white paper with recommendations that dosing devices with numeric graduations and millilitre (mL) units corresponding to labelling should be provided each time oral liquid prescription medications are dispensed. Additionally, dosing devices are recommended to be of appropriate volume and graduated accuracy for the amount prescribed [16].

Most studies on dosing devices describe dosing accuracy by caregivers [15, 17, 18]. In terms of dosing by caregivers, previous research has generated varied conclusions on measuring spoon accuracy. While Yin et al. [19] concluded that the mean dose measured with the calibrated spoon was greater than that of the syringe, Beckett et al. [9] found that the mean volume measured using the 5 mL calibrated spoon was not significantly different from the expected volume. A study by Ellerbeck et al. [20] revealed that 38% of caregivers using calibrated spoons underdosed children over one year old by ≥ 30%, while another study by Kairuz et al. [21] found calibrated spoons recording a mean overdose of 11.8%. A similar trend in dosing accuracy results by caregivers is observed in the literature for other dosing devices [9]. It is worth noting that regardless of caregiver expertise in the use of dosing devices, wrong medication volumes will be measured when inaccurate dosing devices are used. Sadly, recent studies to assess the intrinsic accuracy of dosing devices without the direct influence of caregivers are uncommon and yet necessary [22–24].

In an effort to assess improvements in adherence to industry guidelines, this study systematically evaluates the prevalence and intrinsic accuracy of dosing devices packaged with oral liquid medications in the Ho municipality of Ghana. This study aims to initiate communication by stakeholders with a role in healthcare delivery, manufacturing, and regulation to drive policy on the use of standardized dosing devices for all oral liquid medications. This will potentially assist in overcoming dosing device-related medication errors.

## 2. Materials and Methods

### 2.1. Selection of Community Pharmacies

Eight community pharmacies were randomly selected from a total of seventeen in the Ho municipality. Facilities in operation for a minimum of 3 years were considered for this study. Duplicate data were avoided by analysing dosing devices from a given product only once.

### 2.2. Materials and Equipment

Dosing devices were obtained from the eight selected community pharmacies. Weights were measured using a 200 g Criacr digital balance (UK-KA12) accurate to 0.01 g. Purified water was used for all measurements as viscosity, surface tension, and fluid density have been shown to have little or no effect on the volume of liquid administered [25].

### 2.3. Data Collection

The details of all oral liquid medicines at the selected pharmacies were recorded. Data collected for each product were batch number, product name, product manufacturer, country of origin, drug classification based on the Anatomical Therapeutic Chemical classification system, the need for reconstitution, herbal vs. orthodox, type of dosing device (if available), and volume increments on the dosing device. The accuracy of dosing devices packaged with the products was determined. Measurement of 5 mL and the maximum indicated volume was performed in triplicate for each dosing device.

The initial weights of the empty dosing devices were measured. The dosing devices were then filled with purified water until the bottom of the meniscus was just above the calibration markings. The difference in weight between the empty and filled dosing devices, *m*, was determined and recorded. The volume of water in the filled dosing device was calculated by the relation *v*=*m*/*ρ*, where *v* is the volume of water and *ρ* is the density of purified water at 30°C (∼1.0 gmL^−1^). After measurements were taken, the dosing devices were thoroughly dried, cleaned, and restored to their respective packaging.

The accuracy of each dosing device was determined after the deviation of measured volumes from indicated volumes was computed using the United States Pharmacopoeia (USP) criteria. The USP classifies graduated components as dosing cups, dosing spoons, medicine droppers, and oral syringes [26]. The packaging requirements of the USP categorically stipulate that “Under expected conditions of use, the volume error incurred in measuring liquids for individual dose administration by means of such graduated components should be not more than 10% of the indicated amount of the liquid preparation with which the graduated component will be used” [26]. Hence, in the present study, dosing devices that deviated by more than 10% from the expected volume were considered to have failed the test for accuracy. Percentage deviation was calculated using the absolute value of the relation.

(1) $$\begin{array}{c} \%\;deviation=\left(\frac{Measured\;volume-Expected\;volume}{Expected\;volume}\right)\times 100\text{.} \end{array}$$

### 2.4. Data Analysis

Data have been expressed as mean ± standard deviation. GraphPad Prism (version 9; GraphPad Software, Inc., La Jolla, CA, USA) was used for data analysis. The Wilcoxon matched-pairs signed rank test and analysis of variance (ANOVA) + post hoc Dunnett's test were used to assess differences in measured volumes. *P* values < 0.05 were considered statistically significant for all analyses.

## 3. Results

### 3.1. Classification of Products and Dosing Devices

**Figure 1:**
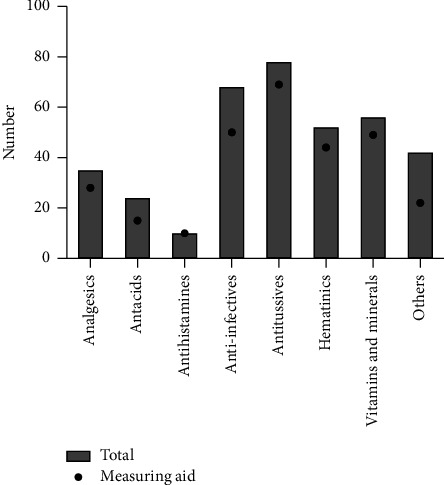
Availability of dosing devices packaged with various categories of oral liquid medicines.

Data were collected for 365 oral liquid preparations from eight randomly selected pharmacies in the Ho municipality. Of the 365 products studied, 78 (21.4%) were not provided with any dosing device; whereas 240 (65.8%), 41 (11.2%), 4 (1.1%), and 2 (0.6%) were packed with cups, spoons, droppers, and syringes, respectively. Details of the dosing devices studied are presented in Table 1. Majority of the products were antitussives (21.4%), followed by anti-infectives (18.6%), vitamins and minerals (15.3%), haematinics (14.2%), analgesics (9.6%), antacids (6.6%), and antihistamines (2.7%). A total of 42 (11.5%) products had less than 10 units each in their respective classes and were therefore categorised as “others.” These products include nootropic agents, urine alkalinators, mucoprotectants, laxatives, colon cleansers, antiasthmatics, anti-colics, antiemetics, antipsychotics, and herbal mixtures with multiple uses. Encouragingly, all antihistamines had dosing devices followed by 88.5%, 87.5%, 84.6%, 80%, 73.5%, 62.5%, and 52.4% of antitussives, vitamins and minerals, haematinics, analgesics, anti-infectives, antacids, and “others,” respectively (Figure 1).

**Figure 2:**
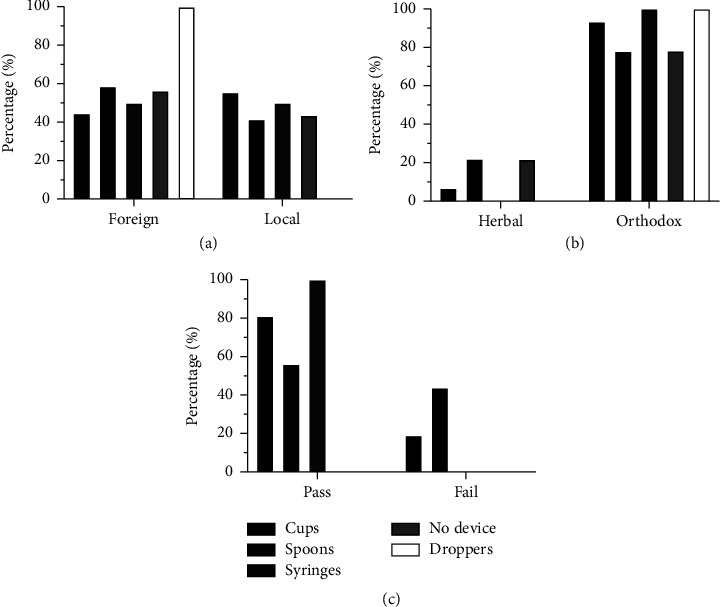
Percentages of dosing devices in terms of (a) country of manufacture, (b) herbal vs. orthodox, and (c) accuracy test according to the USP.

Of the 365 products, 24 (6.6%) were available as powders for reconstitution before use; there were 79.1% anti-infective agents, 4.2% each of antacids and antitussives, and 12.5% nutritional supplements. 4 out of the 24 products which required reconstitution had no measuring aid, even though measurement by the caregiver or dispenser is often a requirement for reconstitution. The results reveal that out of the 365 products studied, 183 (50.1%) were locally manufactured, while 182 (49.9%) were foreign products (i.e., manufactured outside Ghana). 157 (86.2%) foreign products and 166 (90.7%) local products were orthodox preparations. All the products packaged with droppers and 56.4% of products without dosing devices were imported (foreign). Additionally, 58.5% spoons, 50% syringes, and 44.6% cups were packaged with foreign products (Figure 2(a)). Out of 182 foreign products, there were 22% antitussives, followed by 19.8% vitamins and minerals, 15.9% haematinics, 12.6% anti-infectives, and 8.2% antacids. 3.8% each of analgesics and antihistamines were foreign products, while 13.7% of foreign products were categorised as “others” (Table 1). As indicated in Table 1, the order of decreasing percentage of foreign products based on drug class was antihistamines > vitamins and minerals > antacids > haematinics > antitussives > anti-infectives and analgesics. “Others” was comprised of 59.5% foreign products.

### 3.2. 5 mL Accuracy

The minimum and maximum volumes indicated on the dosing devices were 0.10 mL and 30 mL, respectively. Volume increments ranged from 0.10 to 10 mL. An evaluation of the measurement accuracy of 5 mL purified water for all the dosing devices was made except for 8 dosing cups and the 4 droppers which had no 5 mL mark. This is because 5 mL was the most common volume marking among all the dosing devices. As represented in Figure 2(c), 80.7%, 56.1%, and 100% of the cups, spoons, and syringes, respectively, passed the USP criteria for measuring 5 mL of water. 15.9% and 97.7% of the failed cups were packaged with anti-infectives and orthodox preparations. Interestingly, 93.8% of all herbal preparations provided with cups passed the USP test. 38.6% of the failed measuring cups deviated by more than ±15% of 5 mL. Unfortunately, 60% of the spoons that were supplied with anti-infectives failed the USP test for accuracy. 83.3% and 33.3% of the total number of failed measuring spoons were packaged with orthodox preparations and anti-infectives, respectively. In terms of accuracy of cups, there was a marginal difference between locally manufactured and foreign products; this was unlike the results for spoons, where only 38.9% of those packaged with locally manufactured medicines failed the USP test compared to 61.1% for foreign products. Shockingly, 72.2% of the failed measuring spoons deviated by more than ±15% of 5 mL. None of the syringes failed the test for volume accuracy.

### 3.3. Relationship between the Measured Volume for Cups, Spoons, and Syringes

**Figure 3:**
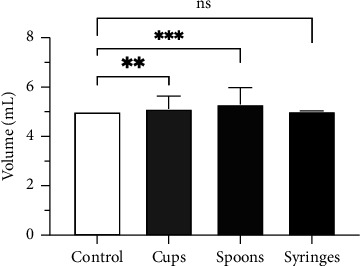
One-way ANOVA + post hoc Dunnett's test of measured volume at the 5 mL mark. Data are expressed as measured volume ± standard deviation, *n* = 3. Statistically significant differences are marked with asterisks ((ns) *p* < 0.12; (*∗∗*) *p* < 0.002; (*∗∗∗*) *p* < 0.001).

In order to assess the differences between the means of the measured 5 mL volumes between cups, spoons, and syringes, a one-way ANOVA + post hoc Dunnett's test was performed. The ANOVA results reveal statistically significant differences in the measured 5 mL volume between cups, spoons, and syringes (*F* (3, 503) = 7.88, *p* < 0.001). As shown in Figure 3, Dunnett's post hoc test revealed that the average volumes measured with the cups and spoons were statistically significantly different from the expected 5 mL (5.14 ± 0.52 mL, *p*=0.006 and 5.3 ± 0.67 mL, and *p* < 0.001, respectively); thus, we reject the null hypothesis that there is no difference between the means. There was no statistically significant difference between the volume measured with the syringes and the control volume (5.01 ± 0.02 mL, *p* > 0.999).

### 3.4. Maximum Volume Accuracy

**Figure 4:**
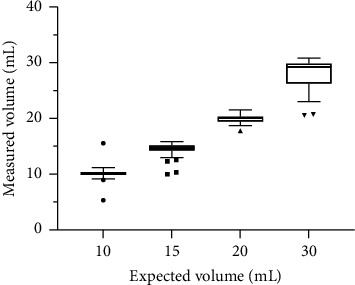
Tukey box and whisker plot of expected vs. measured maximum indicating the volume of dosing cups (*n* = 167, 38, 16, and 17 for 10 mL_max_, 15 mL_max_, 20 mL_max,_ and 30 mL_max_, respectively).

Dosing devices with markings greater than 5 mL were tested for the accuracy of their maximum volume. All syringes and spoons in this study did not have markings for volumes larger than 5 mL. Measuring cups with markings of 10 mL, 15 mL, 20 mL, and 30 mL as their maximum volume recorded 10.14 ± 0.70 mL, 14.40 ± 1.29 mL, 19.86 ± 0.87 mL, and 27.69 ± 3.28 mL, respectively, at the mark. As indicated in Figure 4, cups with a maximum volume of 10 mL (10 mL_max_) had a minimum of 5.33 mL, 9.86 mL 25th percentile, 10.08 mL median, 10.39 mL 75th percentile, and 15.56 mL maximum. For the 15 mL-maximum cups (15 mL_max_), the minimum, 25th percentile, median, 75th percentile, and maximum measured volumes were 9.99 mL, 14.20 mL, 14.70 mL, 15.17 mL, and 15.86 mL, respectively. Cups with 20 mL as the maximum value (20 mL_max_) recorded 17.79 mL, 19.40 mL, 20.03 mL, 20.33 mL, and 21.55 mL as the minimum, 25th percentile, median, 75th percentile and maximum, whilst those with 30 mL as the maximum (30 mL_max_) had 20.56 mL, 26.24 mL, 29.26 mL, 29.86 mL, and 30.85 mL, respectively. Figure 4 indicates the presence of 3, 4, 1, and 2 outliers in the Tukey box and whisker plots for 10 mL_max_, 15 mL_max_, 20 mL_max,_ and 30 mL_max_, respectively; the outliers represent 1.8%, 10.5%, 6.3%, and 11.8% of the measuring cups evaluated for each volume. The difference between maximum and minimum measured volumes decreased in the order 30 mL_max_ > 10 mL_max_ > 15 mL_max_ > 20 mL_max_. Further, the order of decreasing interquartile range, an indicator for the data's variability, was 30 mL_max_, 15 mL_max_, 20 mL_max,_ and 10 mL_max_. Wilcoxon matched-pairs signed rank tests yielded *p* values of <0.001, 0.006, 0.89, and 0.001 for 10 mL, 15 mL, 20 mL, and 30 mL, respectively.

## 4. Discussion

The medication class is worth considering for dose accuracy of medications with a narrow therapeutic window. For example, recent studies have revealed that the in-vitro acid-neutralizing capacities of antacids were within regulatory limitations even after the dose was halved; therefore, it is possible to achieve the desired therapeutic outcome for antacids after halving their doses [27, 28]. Unfortunately, the same cannot be assumed for medications such as antipsychotics, antiemetics, and anti-infectives where overdosing or underdosing may cause the medication to be ineffective or may result in adverse events [29]. Inaccurate administration of anti-infectives can result in the emergence of resistant pathogens [8]. However, 23.1% of the products without a dosing device were anti-infective preparations. It is unacceptable for patients to be burdened with the responsibility of achieving dosing accuracy with their own measuring devices due to the absence of dosing devices from the manufacturer. This is particularly true because of the staggering inaccuracy reported on household dosing devices [6, 8]. As a consequence, critical reconsideration of regulations on dosing device provision in packaging of oral liquid preparations is needed. Majority of the locally manufactured medications were anti-infective products including antibacterial, antifungal, antiviral, and antiparasitic agents such as antimalarials. This is unsurprising due to the overwhelming burden of infectious diseases in Africa [30]. The number of anti-infectives that require reconstitution before use is noticeably large because these products typically undergo aqueous degradation [31].

The results show improvement in the availability of dosing devices provided with herbal preparations since the United States Food and Drugs Administration (USFDA) recommendations in 2009 [13]; 40.5% and 18.9% of herbal and orthodox preparations, respectively, had no dosing devices (Figure 2(b)) compared to the approximately 97% and 46% reported by Bayor et al. [6]. Additionally, none of the herbal preparations were packaged with syringes and droppers (Figure 2(b)). It is estimated that 80% of the world's population use herbal medications in some capacity within their primary healthcare [32]. As the global use of herbal medicines continually grows, there is a need to enforce regulations for the inclusion of dosing devices.

As indicated by Brown et al. [25], viscosity (from 9.8 cP to 47.1 cP), surface tension, and fluid density (between 4 and 40°C) show no effect on the measured volume of liquids in a laboratory setting. ANOVA tests were performed using the results of Brown et al. [25] to evaluate differences in measured volumes of the various liquids, namely, Arovit, nifedipine, Rivotril, propylene glycol, and PEG 200 (Figure SI1(a)). No statistically significant differences in measured volume were observed for the different liquids in the study by Brown et al. [25] using the Arovit dropper (*F* (4, 5) = 0.01127, *p* = 0.9997). In addition, Tukey's multiple comparison tests on all pairs of liquids showed no statistically significant differences (Table SI1). Secondly, Elliot et al. [22] evaluated the effect of viscosity and density of liquid acetaminophen formulations on the volume measured using a syringe, dropper, and cup in a laboratory setting. The liquid acetaminophen formulations were prepared with grape suspension, cherry suspension, and cherry solution as the continuous phase. The order of decreasing viscosity of the formulations was grape suspension (1240 cP), cherry suspension (1090 cP), and cherry solution (51.4 cP). ANOVA tests using the results of Elliot et al. [22] to assess differences in measured volume of the acetaminophen formulations reveal that although the acetaminophen formulations ranged in viscosity from 51 to 1240 cP, there were no statistically significant differences in volume measured (*F* (2,6) = 1.37, *p* = 0.32) (Figure SI1(b)). Tukey's multiple comparison tests revealed no statistically significant differences between the measured volume of grape suspension versus cherry suspension (*p* = 0.96) and cherry suspension versus cherry solution (*p* = 0.46) (Table SI2). Intriguingly, grape suspension and cherry solution which differed in viscosity by approximately 1200 cP also showed no statistically significant difference in measured volume (*p* = 0.33) (Table SI2). Brown et al. [25] and Elliot et al. [22] did not emphasize the accuracy of measuring water with dosing devices. However, the encouragingly similar volumes measured over a wide viscosity range clearly suggest that in a laboratory setting, precise evaluation of intrinsic volume accuracy of dosing devices can be achieved for liquids with viscosity up to 1240 cP [22, 25]. According to the results of Brown et al. [25] and Elliot et al. [22], volume accuracy was influenced by the viscosity of the liquid outside a laboratory setting. Therefore, purified water was used as the model liquid in the present study. It was not indicated on any product in the present study that its dosing device must be used to measure only that product. This means that a caregiver or patient may use a dosing device packaged with a product to measure another liquid medicine. This study was limited to an assessment of whether the different devices could be used to accurately measure a model liquid, and by extension, the products they were packaged with and any other liquid medicine.

Based on the results of the present study, oral syringes are the most accurate dosing device for measuring 5 mL according to the USP, followed by cups, and spoons. The standard deviation of dosing devices was determined by Beckett et al. [9] and Sobhani et al. [33] in order to describe the range of volumes caregivers measure with each device; it was established that the smaller the standard deviation, the more consistent dosing by a caregiver is. The standard deviation of the oral syringe in the present study (±0.02 mL) is considerably lower than the ±0.35 mL and ±0.7 mL quoted by Beckett et al. [9] and Sobhani et al. [33], respectively. Even the spoons, which were the least accurate in the present study, had a lower standard deviation (±0.67 mL) than the results reported by Beckett et al. [9] (±0.76 mL). This result suggests that the full potential of dosing devices may not have been completely exploited by the caregivers in the research by Beckett et al. [9] and Sobhani et al. [33]. Alternatively, the discordance between the results in the present study and the literature could be a result of improvement in the inherent accuracy of dosing devices; this is unlikely because it recently has been established in practice that dosing volume accuracy varies among caregivers regardless of the accuracy of the dosing device [9, 15]. These findings highlight, in addition to having accurate dosing devices, the need for improved caregiver education regarding the use of different dosing devices. Deviation by more than ±15% of 5 mL means that for a 5 mL dose of drug, an overdose or underdose of more than 30 mg of the drug could be administered if the amount of active ingredient in the medication is 200 mg/5 mL. Deviation of 38.6% of the failed measuring cups and 72.2% of the failed measuring spoons by more than ±15% of 5 mL is undesirable, particularly for medications that require frequent dosing and are commonly associated with adverse events.

Clearly, the least accurate measuring cup in terms of maximum volume accuracy was 30 mL_max_; this is because coupled with the 10.2 mL difference between the measured maximum and minimum volumes, the interquartile range for 30 mL_max_ was the largest. Wilcoxon matched-pairs signed rank tests, nonparametric hypothesis tests, were used to compare the measured maximum volumes of the measuring cups to their maximum indicated volumes. Based on the *p* values, we reject the null hypothesis that there is no difference between the measured maximum volumes and the expected volumes for 10 mL_max_, 15 mL_max_, and 30 mL_max_. Large magnitudes of the outliers influenced the results of the Wilcoxon test, particularly for 10 mL_max_ and 15 mL_max_ which had 66.7% and 50%, respectively, of the outliers deviating by at least ±50% of the expected volume. This may be accountable for the apparent inaccuracy observed for the 10 mL_max_ and 15 mL_max_ cups. The accuracy of the cups with a maximum volume of 20 mL is confirmed by the large *p* value; there is no statistically significant difference between the measured volumes and the control volume.

Cups have been associated with more than three times higher likelihood of caregiver-based error than oral syringes; specifically, this is true for small-dose volumes. Caregivers may find cups to be inherently difficult to use because the entire cup may be wrongly considered to be the dose, the cup may not be placed on a level surface while measuring, or the markings and meniscus of measure may not be observed at eye level [15]. Although the syringe has been determined to be the most accurate dosing device in this study, using a 5 mL syringe to dispense a 7.5 mL dose will result in the need to fill the syringe multiple times, which will rely heavily on caregiver numeracy skills. Therefore, large-volume dosing devices must be supplied or packaged with oral liquid medicines that are taken in large volumes. Additionally, it is preferred if manufacturers ensure that dosing devices that accompany oral liquid medicines have all the relevant volume markings that correspond to indicated doses on their products. This will help prevent multiple uses of a device per dose administration and can further reduce or prevent errors in dose measurement. Apart from these dosing device-related recommendations, it is essential to explain dose volumes to caregivers whenever an oral liquid medicine is dispensed as it cannot be assumed that caregivers are capable of reading and interpreting the markings on dosing devices. Caregivers must also be taught how to correctly use oral syringes, as these dosing devices require additional skills for use compared to cups and spoons. For example, when using an oral syringe, the caregiver must ensure that the tip of the syringe remains in the liquid to avoid drawing air into the syringe. After the right dose has been taken, the syringe must be aimed at the area between the gums and the inside of the cheek to administer the medication in bits.

## 5. Conclusion

Variations in measured volumes of medicines may result in underdosing or overdosing, which can have dire consequences, particularly for medicines with narrow therapeutic windows. This study sought to determine the prevalence and accuracy of dosing devices packaged with oral liquid medications in the Ho municipality of Ghana. While the study is specific to the Ho municipality of Ghana, there are significant aspects of it which have been highlighted that are relevant globally. For example, a measuring device was provided for 78.6% of the products surveyed. Spoons had the lowest volume accuracy according to the USP criteria and the highest deviation of ±15% from the target value. Antacids were the least likely to have a dosing device followed by anti-infectives, analgesics, haematinics, vitamins and minerals, and antitussives. Further, syringes were found to be the most accurate but the least available dosing device. Accurate dosing of anti-infective medications is of paramount importance. However, the majority of the spoons were packaged with anti-infectives. Measuring cups and spoons packaged with herbal preparations were significantly less likely to fail the test for volume accuracy compared to orthodox preparations. The availability and accuracy of dosing devices for oral liquid medications remain inadequate despite the USFDA recommendations. The results of this study highlight the need for advocacy on the provision of the oral syringe as the dosing device of choice for all manufacturers of oral liquid medications in order to decrease the likelihood of dosing errors.

## Figures and Tables

**Table 1:** Categories of oral liquid medicines studied.

Products	Number of products (%)^*∗*^				
	Total	Country of origin		Dosing device	
		Local	Foreign	Available	Not available
Analgesic	35 (9.6)	28 (80)	7 (20)	28 (80)	7 (20)
Antacid	24 (6.6)	9 (37.5)	15 (62.5)	15 (62.5)	9 (37.5)
Antihistamine	10 (2.7)	3 (30)	7 (70)	10 (100)	0 (0)
Anti-infective	68 (18.6)	45 (66.2)	23 (33.8)	50 (73.5)	18 (26.5)
Antitussive	78 (21.4)	38 (48.7)	40 (51.3)	69 (88.5)	9 (11.5)
Haematinic	52 (14.2)	23 (44.2)	29 (55.8)	44 (84.6)	8 (15.4)
Vitamins and minerals	56 (15.3)	20 (35.7)	36 (64.3)	49 (87.3)	7 (12.5)
Others	42 (11.5)	17 (40.5)	25 (59.5)	22 (52.4)	20 (47.6)

^*∗*^(%) for “Total” represents a percentage of the total number of products studied. (%) for country of origin and availability of dosing device represent a percentage of the total of each individual product category.

## Data Availability

The data used to support the current study are available from the corresponding author upon request.

## Supplementary Materials

Supplementary Materials Figure SI1 shows the results of one-way ANOVA tests of dosing device volume accuracy for data extracted from Brown et al. [25] (A) and Elliot et al. [22] (B). While Brown et al. [25] studied the volume accuracy of droppers on Arovit, nifedipine, Rivotril, propylene glycol, and PEG 200, Elliot et al. [22] compared the dosing accuracy of syringes, droppers, and cups for paracetamol preparations with cherry solution, cherry suspension, and grape suspension as the continuous phase. No statistically significant differences in measured volume were observed for the different liquids in both studies [22, 25]. Table SI1 indicates Tukey's post hoc multiple comparison test results for dosing device volume accuracy on data extracted from Brown et al. [25]. All the comparisons showed no statistically significant differences in measured volume. Table SI2 shows the results of Tukey's post hoc multiple comparison for dosing device volume accuracy on data extracted from Elliot et al. [22]. All the comparisons showed no statistically significant differences in measured volume.
