# Supplementary material for: Assessment of the Availability and Accuracy of Dosing Devices Packaged with Oral Liquid Medications in the Ho Municipality of Ghana
Source: Scientifica (Cairo). 2022 Oct 20;2022:9223858. doi: 10.1155/2022/9223858 (PMC9613386; doi:10.1155/2022/9223858)
Supplement: Supplementary Materials — Figure SI1 shows the results of one-way ANOVA tests of dosing device volume accuracy for data extracted from Brown et al. [25] (A) and Elliot et al. [22] (B). While Brown et al. [25] studied the volume accuracy of droppers on Arovit, nifedipine, Rivotril, propylene glycol, and PEG 200, Elliot et al. [22] compared the dosing accuracy of syringes, droppers, and cups for paracetamol preparations with cherry solution, cherry suspension, and grape suspension as the continuous phase. No statistically significant differences in measured volume were observed for the different liquids in both studies [22, 25]. Table SI1 indicates Tukey's post hoc multiple comparison test results for dosing device volume accuracy on data extracted from Brown et al. [25]. All the comparisons showed no statistically significant differences in measured volume. Table SI2 shows the results of Tukey's post hoc multiple comparison for dosing device volume accuracy on data extracted from Elliot et al. [22]. All the comparisons showed no statistically significant differences in measured volume. [file 9223858.f1.docx]

**SUPPLEMENTARY INFORMATION**

**Assessment of the Availability and Accuracy of Dosing Devices Packaged with Oral Liquid Medications in the Ho Municipality of Ghana**

Yussif Saaka^1*^, David M. Nyamadi^1^, Hilda Amekyeh^1^, Adelaide Mensah^1^

^1^Department of Pharmaceutics, School of Pharmacy, University of Health and Allied Sciences, PMB 31, Ho, Ghana

^*^Correspondence should be addressed to Yussif Saaka; ysaaka@uhas.edu.gh

**Figure SI1**. Results of one-way ANOVA tests of dosing device volume accuracy on data extracted from Brown et al.[25] (A) and Elliot et al.[22] (B).

**Table SI1**. Tukey’s post-hoc multiple comparison test results for volume accuracy on data extracted from Brown et al.[25]

|  | Mean  difference | 95% CI of  difference | Adjusted p-value |
| --- | --- | --- | --- |
| Arovit vs. Nifedipine | 0.0005 | -0.3832 to 0.3842 | >0.9999 |
| Arovit vs. Rivotril | 0.0125 | -0.3712 to 0.3962 | >0.9999 |
| Arovit vs. PEG 200 | 0.005 | -0.3787 to 0.3887 | >0.9999 |
| Arovit vs. Propylene glycol | 0.016 | -0.3677 to 0.3997 | 0.9998 |
| Nifedipine vs. Rivotril | 0.012 | -0.3717 to 0.3957 | >0.9999 |
| Nifedipine vs. PEG 200 | 0.0045 | -0.3792 to 0.3882 | >0.9999 |
| Nifedipine vs. Propylene glycol | 0.0155 | -0.3682 to 0.3992 | 0.9998 |
| Rivotril vs. PEG 200 | -0.0075 | -0.3912 to 0.3762 | >0.9999 |
| Rivotril vs. Propylene glycol | 0.0035 | -0.3802 to 0.3872 | >0.9999 |
| PEG 200 vs. Propylene glycol | 0.011 | -0.3727 to 0.3947 | >0.9999 |

**Table SI2**. Tukey’s post-hoc multiple comparison test results for volume accuracy on data extracted from Elliot et al.[22]

|  | Mean  difference | 95% CI of  difference | Adjusted p-value |
| --- | --- | --- | --- |
| Grape suspension vs. Cherry suspension | -0.09 | -1.065 to 0.8850 | 0.9571 |
| Grape suspension vs. Cherry solution | -0.4933 | -1.468 to 0.4817 | 0.3343 |
| Cherry suspension vs. Cherry solution | -0.4033 | -1.378 to 0.5717 | 0.4604 |

**References**

[22] Elliott JP, McConaha J, Cornish N, Bunk E, Hilton L, Modany A, et al. Influence of Viscosity and Consumer Use on Accuracy of Oral Medication Dosing Devices. J Pharm Technol 2014;30. https://doi.org/10.1177/8755122514533780.

[25] Brown D, Ford JL, Nunn AJ, Rowe PH. An assessment of dose-uniformity of samples delivered from paediatric oral droppers. J Clin Pharm Ther 2004;29. https://doi.org/10.1111/j.1365-2710.2004.00595.x.
